# Functionalization of electrospun fish gelatin mats with bioactive agents: Comparative effect on morphology, thermo‐mechanical, antioxidant, antimicrobial properties, and bread shelf stability

**DOI:** 10.1002/fsn3.2676

**Published:** 2021-12-10

**Authors:** Kaiser Mahmood, Hanisah Kamilah, Abd Karim Alias, Fazilah Ariffin, Abdorreza Mohammadi Nafchi

**Affiliations:** ^1^ Food Technology Division School of Industrial Technology Universiti Sains Malaysia Penang Malaysia; ^2^ Department of Crop Science, Faculty of Agriculture and Forestry Universiti Putra Malaysia Bintulu Sarawak Campus Bintulu Malaysia; ^3^ Halal Products Research Institute Universiti Putra Malaysia Serdang Malaysia; ^4^ Department of Food Science and Technology, Damghan Branch Islamic Azad University Damghan Iran

**Keywords:** active packaging, antifungal, bread, electrospinning, fish gelatin, limonene

## Abstract

In the current study, fish gelatin‐based nanofiber mats were embedded with different bioactive agents (BAs) such as cinnamaldehyde (CEO), limonene (LEO), and eugenol (EEO) at 1, 3, and 5% *via* electrospinning, and their effects on the morphological, structural, mechanical, thermal, antioxidant, antimicrobial, and bread packaging properties of the mats were evaluated. The gelatin mats presented different physicochemical properties due to the inherent differences in the chemical structure of the added BAs and their interaction with the gelatin chains. The conductivity, surface tension, and viscosity of gelatin dopes changed with the presence of the BAs, yet the electrospun nanofibers showed defect‐free uniform morphology as confirmed by electron microscopy, with no significant change in the chemical structure of gelatin. The melting temperature of gelatin mats remained in the range of 187–197°C. The mats presented lower tensile strength and elongation at break by the addition of BAs compared with the pristine gelatin mat. The highest radical scavenging (90%) was yielded by mats with EEO, while mats with CEO depicted better antibacterial activity with an inhibition zone of 18.83 mm. However, a dose‐dependent increase in the antifungal properties was noticed for all the mats. The mats retained almost 50% of BAs after 60 days of storage at 45% relative humidity. Electrospun gelatin mats inhibited the aerobic bacteria (81%) and yeast and molds (61%) in preservative‐free bread after 10 days of storage.

## Graphical abstract

Fish gelatin‐based nanofiber mats were embedded with bioactive agents (BAs) such as cinnamaldehyde (CEO), limonene (LEO), and eugenol (EEO) at 1, 3, and 5% via electrospinning. The BAs added in the mats being different in the chemical structure were interacted differently with gelatin and manipulated the gelatin mat properties. Electrospun gelatin mats presented a good inhibition of aerobic bacteria (81%), and yeast and molds (61%) in preservative‐free bread after 10 days of storage.

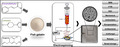

## 1 INTRODUCTION

To feed an ever‐increasing population, food spoilage should be minimized. Microbial proliferation on the food surface is one of the principal reasons of food spoilage. At the same time, addition of antimicrobial preservatives causes food components to lose their effectiveness besides compromising the food organoleptic properties. Thus, the development of active packaging by incorporating antimicrobial preservatives in the polymeric carrier is a suitable alternative where the polymer matrix acts as a reservoir and tailors the release of these bioactive agents (BAs; Tampau et al., 2017). The diverse availability of synthetic plastics makes them an attractive carrier matrix for developing active packaging. However, the lack of biodegradability and hazardous components (monomers and plasticizers) are posing intense environmental risk. Moreover, the generation of a huge mass of refuse threatens the safety of food and the ecosystem (Chen et al., 2019).

In this regard, biopolymers are suitable alternatives, and being nontoxic and sustainable, they are used overwhelmingly to fabricate biodegradable active packaging. Typically, proteins with inherently good mechanical and better gas‐barrier properties have been evaluated in active packaging (Altan et al., 2018; Oladzadabbasabadi et al., 2022). Gelatin, an animal protein, is a low‐cost and abundant raw material and has been tested in developing biodegradable active packaging (Acosta et al., 2016; Mele, 2020). The major sources of gelatin are bovine and porcine skin; however, immunological and ethno‐religious concerns have shifted the priority toward fish gelatin. Good emulsification, biodegradability, and biocompatibility make gelatin a better polymeric carrier for various BAs. Moreover, abundant polar and nonpolar functional groups bind and interact with different BAs resulting in their better encapsulation and retention in the gelatin matrix (Acosta et al., 2016; Lin et al., 2018).

Essential oils (EOs) are natural BAs found in plants that have been widely employed in developing antimicrobial active packaging systems (Acosta et al., 2016; Mele, 2020). EOs are plant secondary metabolites, which include terpenes, phenols, aldehydes, alcohols, etc. They are generally recognized as safe (GRAS) with well‐known antimicrobial and antioxidative characteristics. Based on the hydrophobic nature and polarity, EOs improve antimicrobial and antioxidant properties of the polymeric packaging matrix. (Burt, 2004; Mele, 2020).

Conventional techniques of developing active packaging materials usually involve simple dispersion or emulsification of hydrophobic BAs in the bulk polymeric matrix. The higher processing temperature, the limited surface area of the developed matrix, and thermodynamic incompatibility between hydrophobic BAs and biopolymer chains lead to non‐negligible losses of BAs by steam drag effect (Altan et al., 2018; Tampau et al., 2017). Recently, electrospinning has been reported in developing active packaging mats by encapsulating EOs, vitamins, etc. (Ahmadi et al., 2021; Lin et al., 2018). It is a straightforward, cost‐effective, and simple technique for developing nanomaterials compared with other methods, such as self‐assembly, nanolithography, and melt fibrillation (Mele, 2020).

Electrospinning produces nonwoven mats comprised of micro‐ to nano‐scale fibers under high electrostatic potential. The submicron diameter, high surface area, and porosity of fibers make electrospinning the method of choice (Haider et al., 2018). Typically, the embedding of BAs into microfibers or nanofibers improves their bioactivity, stability, and solubility, and impedes the premature interaction with food or package headspace (Tonyali et al., 2020). Recently, various studies presented the incorporation of BAs such as plant extracts and EOs in electrospun polymeric films and reported enhanced antioxidant and antimicrobial properties of the films (García‐Salinas et al., 2020; Haghighi et al., 2019; Mele, 2020). Haghighi et al. (2019) employed EOs from cinnamon, pink clove, nutmeg, thyme, and citronella in chitosan‐gelatin films by solvent casting as active packaging and reported good in vitro antibacterial activity. Similarly, Tonyali et al. (2020) incorporated thymol, eugenol, and cinnamic aldehyde in pullulan‐based active packaging and studied the thermal properties and release behavior of the bioactive compounds. Recently, electrospun gelatin films were functionalized by cinnamon extract and chitosan; however, only structural, thermal, and in vitro antibacterial properties were reported (Ahmadi et al., 2021).

To the authors’ knowledge, the use of electrospun fish gelatin mats as active packaging for preservative‐free wheat bread has not been reported yet. Moreover, structure‐dependent bioactivities of different BAs in fish gelatin‐based electrospun active mats have not been investigated thoroughly. Thus, in the current study, three BAs, such as cinnamaldehyde (CEO; an aldehyde), limonene (LEO; a terpene), and eugenol (EEO; a phenol derivative), were embedded in fish gelatin mats *via* electrospinning, and their effect on the morphological, structural, and functional properties of the mats was estimated. The three selected bioactive compounds with different chemical structures and hydrophobicity were expected to improvise the antioxidant and antimicrobial properties of electrospun fish gelatin mats and potentially extend the shelf stability of preservative‐free wheat bread.

## 2 MATERIALS AND METHODS

### 2.1 Chemicals and reagents

Fish gelatin type‐A (Bloom number 210 g) was obtained from SIM Company (Penang, Malaysia). Gas chromatographic grade BAs, cinnamaldehyde (CEO: 99.5%), limonene (LEO: 99.8%), and eugenol (EEO: 99.7%), were obtained from R & M Chemicals (UK). Analytical grade glacial acetic acid, hydrochloric acid, ethanol, and methanol were procured from QRec Chemicals (Malaysia). Free radical, 2,2‐diphenyl‐1‐picrylhydrazyl (DPPH), and 2,4,6‐tripyridyl‐s‐triazine (TPTZ) were purchased from Sigma (USA). Ferric chloride hexahydrate (FeCl_3_.6H_2_O), ferrous sulfate (Fe_2_SO_4_), and sodium acetate trihydrate were supplied by Merck Millipore (USA). Nutrient broth, nutrient agar, Mueller‐Hinton agar, and potato dextrose agar were bought from HiMedia (India).

### 2.2 Spinning dope preparation

Spinning dope of fish gelatin (30% w/v) was prepared in acetic acid (30% v/v) by stirring the preweighed gelatin powder at 50°C for 6 h according to Mahmood et al. (2019). The CEO, LEO, and EEO were homogenized in the gelatin dope at 1, 3, and 5% w/w of the fish gelatin at 40°C for 6 h.

### 2.3 Spinning dope characterization

The electrical conductivity (mS/cm) of gelatin dope was measured by using a waterproof conductivity meter (sensION 5, Hach 51800‐10, Cole‐Parmer). The dope was filled in a 25‐ml glass beaker, and the electrode was equilibrated for 3 s before taking the readings. A semiautomatic tensiometer (Tensiomat^®^, Thermo Fisher Scientific) was used to measure the apparent surface tension (dyne/cm) by the du‐Noüy ring method. The cleaned ring was immersed (1 cm) deep into the dope filled in a 15‐ml glass beaker and slowly removed and detached from the surface of the dope. The viscosity (Pa.s) was measured by a rheometer (AR‐1000–N, TA Instruments Inc.) employing a flat plate geometry (dia 40 mm, gap 1.0 mm) at a shear rate of 0–200 s^−1^. The dope (0.5 ml) was applied on the Peltier plate of the rheometer and equilibrated before shearing. All measurements were made at 25 ± 1°C.

### 2.4 Electrospinning of gelatin dopes

The electrospinning setup was comprised of three main components: (1) a high‐voltage power supply (Spellman CZE100r), (2) a syringe pump (New Era 1000), and (3) a static plate collector. The electrospinning process was carried out at 17 kV, 0.5 ml/h, and 15 cm of voltage, flow rate, and spinneret tip‐to‐collector distance, respectively. Electrospinning was conducted at ambient conditions at 40 ± 2% relative humidity. The electrospun fibers were collected as a mat on a grounded plate collector covered with aluminum foil.

### 2.5 Morphology of electrospun fibers in mats

The shape and diameter of fibers were assessed by using a field emission scanning electron microscope (FESEM). The sample was attached to the stub using a double tape and coated with gold for 60 s at 20‐mA current and pressure of 1 × 10^3^ mbar by a sputter coater (Quorum‐Q150 TS) and photographed at 10,000× magnification by an FEI Quanta *SEM* (FEG‐650, FEI Technologies Inc.) at a voltage of 15 kV. The diameter of six randomly selected fibers was measured and averaged.

### 2.6 Chemical structure analysis by ATR‐FTIR

Structural changes in the electrospun gelatin mat with BAs were assessed using attenuated total reflectance Fourier transform infrared spectrometer (ATR‐FTIR) equipped with a Zn/Se laser (IR Prestige21, Shimadzu). The sample was scanned from 400 to 4000 cm^−1^ at transmittance mode with 64 scans at a resolution of 4 cm^−1^. IR^®^ solution software was used to analyze the spectra of gelatin mats.

### 2.7 Differential scanning calorimetry (DSC)

Thermal properties of electrospun gelatin mats were evaluated by using a calibrated differential scanning calorimeter (DSC‐Q200, TA Instruments) under nitrogen environment, and the thermograms were analyzed by TA‐universal^®^ analysis software to obtain glass transition (*T*_g_) and melting temperature (*T*_m_). The sample (3–5 mg) was weighed in a standard aluminum pan (#900786.901) and sealed using the pan crimper. The sample was heat scanned in the range of 30–250°C with a heating rate of 10°C/min. An empty aluminum pan was used as a reference.

### 2.8 Mechanical properties

The mechanical properties of mats were determined using a tensile tester (EZ‐S‐500N, Shimadzu). The rectangular‐shaped sample (20 mm ×5 mm) was conditioned for 48 h at 40 ± 2% relative humidity at ambient temperature. The sample was prepared by using two layers of graph paper (of 20 mm^2^), and the double tape was used to stick the sample between grips of the tensile tester. The initial separation of grips was maintained at 10 mm, and the test was carried out at a crosshead speed of 10 mm/min. The tensile strength (TS), elongation at break (EAB), and Young's modulus (Y) were calculated from the stress–strain curve.

### 2.9 Antioxidant activity of mats

#### 2.9.1 Radical scavenging activity (RSA)

RSA of the gelatin mat was estimated through the DPPH method (Sutaphanit & Chitprasert, 2014). Briefly, the mat (20 mg) was extracted in 5 ml of 80% methanol and centrifuged at 2300 *g* for 5 min. After precipitation of gelatin, the clear supernatant (150 μl) was mixed with the DPPH reagent (150 μl) and allowed to react at room temperature for 30 min in the dark. The absorbance of the reaction mixture was measured at 517 nm. The RSA was computed by the following equation:

Radicalscavengingactivity(\% )=(AbsDPPH‐Abssample)/AbsDPPH×100

where Abs DPPH and Abs sample represent the absorbance values of DPPH solution and mat‐extracted supernatant.

#### 2.9.2 Ferric reducing antioxidant power (FRAP) assay

The FRAP assay was conducted according to Tongnuanchan et al. (2016) with slight modifications. The FRAP reagent was prepared by mixing acetate buffer, FeCl_3_ solution, and TPTZ (tripyridyltriazine) solution at a 10:1:1 ratio. The supernatant (150 μl) was mixed with 150 μl of the FRAP reagent and thoroughly vortexed. The mixture was incubated at room temperature in the dark for 30 min, and the absorbance was measured at 593 nm. Ferrous sulfate (FeSO_4_.7H_2_O) was used as standard, and the results were expressed as μmol eq Fe^+2^/g mat.

### 2.10 Encapsulation efficiency

Encapsulation efficiency (EE%) of gelatin mats for BAs was estimated by the ratio of FRAP value of nonencapsulated BAs to the encapsulated counterparts according to Torkamani et al. (2018).

### 2.11 In vitro antibacterial activity

Antibacterial activities of mats were tested by the disk diffusion method against a gram‐positive bacterium (*Staphylococcus aureus*) and a gram‐negative bacterium (*Escherichia coli*). Cultures of both the bacterial strains were adjusted to 0.5 McFarland, and 100 μl was swabbed on Mueller‐Hinton agar. Gelatin mat disks (8 mm) were then fixed on to the cultured plates of bacteria and incubated at 37°C for 16–24 h. The growth inhibition zones (mm) for bacteria were measured to observe the antibacterial efficiency of the mats.

### 2.12 In vitro antifungal activity

For evaluation of antifungal activity of gelatin mats, *Aspergilus niger* spores were cultured on potato dextrose agar. The spores were harvested after 3 days using sterilized distilled water, and the density was adjusted equal to 0.5 McFarland. One hundred microliters of the spore culture was poured and swabbed on potato dextrose agar in Petri plates. Circular disks (8 mm) of the gelatin mat were then placed on to the cultured plates and incubated for 72 h at 25°C. The zone of fungal growth inhibition (mm) was measured in triplicate to estimate the antifungal efficiency of gelatin mats.

### 2.13 Functional stability of gelatin mats

Stability of mats was estimated under storage at ambient temperature for 60 days at 45 ± 2% relative humidity. The retention of BAs in the mats was estimated through the FRAP assay (as mentioned in 2.9.2) with an interval of 10 days. The FRAP value was correlated with the amount of active compounds retained within the mats and further converted into the release (%) by subtracting the retention from 100 and plotted as a cumulative release against time (days).

### 2.14 Bread preservation study

The gelatin mats were tested for improving the shelf stability of preservative‐free wheat bread. The following ingredients were added for bread preparation based on the weight percentage of wheat flour: (1) 4% instant dry yeast, (2) 4% of shortening, (3) 8% sucrose, (4) 2% salt, and (5) 60%–64% distilled water. After baking, the bread was cooled before slicing and stored in polyethylene bags for further analysis. The bread crumb (10 ± 0.5 g) was placed in the sterile Petri plate (110 mm × 25 mm), and the mat (40 mg) was placed within the Petri plate without direct contact with bread. The Petri plate was sealed with parafilm and incubated at room temperature for 10 days. A control sample was prepared by using gelatin film without BAs. To estimate the microbial load, the bread sample was homogenized in peptone water (0.1%) in a stomacher at a ratio of 1:10, and serial dilutions were made from 10^−1^ to 10^−5^. Almost 100 μl of the homogenate was poured on nutrient agar and potato dextrose agar for counting total aerobic bacteria, and yeast and molds, respectively. The Petri plates were incubated at 37°C and 25°C for 16–24 h and 72–96 h for counting bacteria, and yeast and molds, respectively. The efficiency of the mat in preserving the bread was determined by the following equation:

Inhibitionefficiency(%)=(CFUcontrolbred‐CFUsamplebred)/CFUcontrolbred×100

where CFU control bread and sample bread indicate the colony‐forming units in bread stored with gelatin mats with and without BAs.

### 2.15 Statistical analysis

Analysis of variance (ANOVA) was performed using SPSS‐23.0 (IBM), and for mean comparison, the Duncan multiple range test (DMRT) was applied. *p* < .05 was considered statistically significant.

## 3 RESULTS AND DISCUSSION

### 3.1 Spinning dope characterization

The effect of BAs on the conductivity, viscosity, and surface tension of gelatin spinning dope is presented in Table 1. Spinning dope properties are crucial in defining the spinning behavior and morphology of the developed fibers. A substantial conductivity of the spinning dope is inevitable to realize the process of electrospinning, as the process is based on electrostatic principles (Haider et al., 2018). Thus, any change in the dope conductivity directly influences the thickness of the developed fibers in the mats. The gelatin is an ampholyte and contains basic and acidic amino acids, which ionize in the presence of acetic acid. The conductivity of gelatin dope was reduced in the presence of BAs, yet it remained in the spectrum where electrospinning was successful. By increasing the CEO from 1% to 5%, dope conductivity was lowered significantly (*p* < .05); however, the dope with LEO presented the least conductivity. This reduction in conductivity could be correlated with the low polarity of the BAs where LEO is least polar due to its terpene‐like nature. Karami et al. (2021) also reported reduced conductivity of dope prepared from chitosan‐flaxseed mucilage added with *Ziziphora clinopodioides* EO.

**TABLE 1:** Properties of gelatin spinning dope with the addition of bioactive agents

Sample	Conc. (%)	Conductivity (μS/cm)	Surface tension (dyne/cm)	Viscosity* (Pa.s)	Fiber diameter (nm)
Control	‐	2014.3 ± 2.02^a^	46.16 ± 0.21^c^	1.234 ± 0.08^a^	314.30 ± 21.8^b^
CEO	1	1654.0 ± 4.00^d^	49.4 ± 0.87^a^	0.823 ± 0.02^e^	358.3 ± 112.7^ab^
	3	1646.0 ± 5.26^e^	45.9 ± 0.10^c^	0.841 ± 0.02^de^	372.6 ± 74.8^ab^
	5	1635.0 ± 2.64^f^	46.6 ± 0.57^bc^	0.854 ± 0.01^cde^	441.0 ± 120.3^a^
LEO	1	1662.7 ± 4.50^c^	47.3 ± 0.37^b^	0.819 ± 0.01^e^	351.3 ± 55.8^ab^
	3	1647.3 ± 2.51^e^	48.6 ± 0.15^a^	0.815 ± 0.01^e^	358.0 ± 26.4^ab^
	5	1621.3 ± 3.21^g^	49.0 ± 0.30^a^	0.803 ± 0.05^e^	446.3 ± 141.5^a^
EEO	1	1654.0 ± 2.64^d^	49.4 ± 0.55^a^	0.888 ± 0.01^bcd^	401.6 ± 55.5^a^
	3	1677.3 ± 4.04^b^	46.6 ± 0.34^bc^	0.907 ± 0.01^bc^	408.3 ± 56.5^a^
	5	1659.0 ± 2.64^cd^	42.5 ± 0.47^d^	0.939 ± 0.01^b^	468.00 ± 133.0^a^

Note

Mean values with same letters are nonsignificant (*p* < .05).

Abbreviations: CEO, cinnamaldehyde; EEO, eugenol; LEO, limonene.

*Viscosity is reported at 100 s^−1^.

Similarly, a reduction in viscosity of spinning dope was seen in the presence of BAs compared with the control, which suggests the reduced intermolecular interaction of gelatin chains. Almost 30%–40% reduction in viscosity was noticed for dopes with BAs. This outcome is in line with a previous study where EO addition resulted in lowering of dope viscosity (Karami et al., 2021). However, increasing the concentration of BAs from 1% to 5% did not increase dope viscosity significantly. Generally, thicker fibers are produced from a viscous dope compared with a thinner or less viscous dope (Haider et al., 2018). All the spinning dopes presented almost Newtonian flow behavior where the flow behavior index remained near to unity (*n* ~ 1; data not shown).

Surface tension is the primary force opposing the applied voltage during the electrospinning process and determines the spinnability of the dopes (Anu Bhushani & Anandharamakrishnan, 2014). Typically, at lower polymer concentration and viscosity, the surface tension in the dominant force impedes the formation of bead‐free uniform nanofibers (Deng et al., 2018). In the presence of BAs, especially at 1%, the surface tension of spinning dopes increased significantly than the control (Table 1). An increasing concentration of BAs to 5% reduced the cohesiveness among solvent molecules at the liquid/air interface and resulted in lower surface tension of the dopes. It is suggested that high surface tension of dope inhibits the process of electrospinning, thereby causing the instability of the polymer jet resulting in the formation of particles instead of fibers (Anu Bhushani & Anandharamakrishnan, 2014).

### 3.2 Morphology of electrospun fibers in mats

**FIGURE 1:**
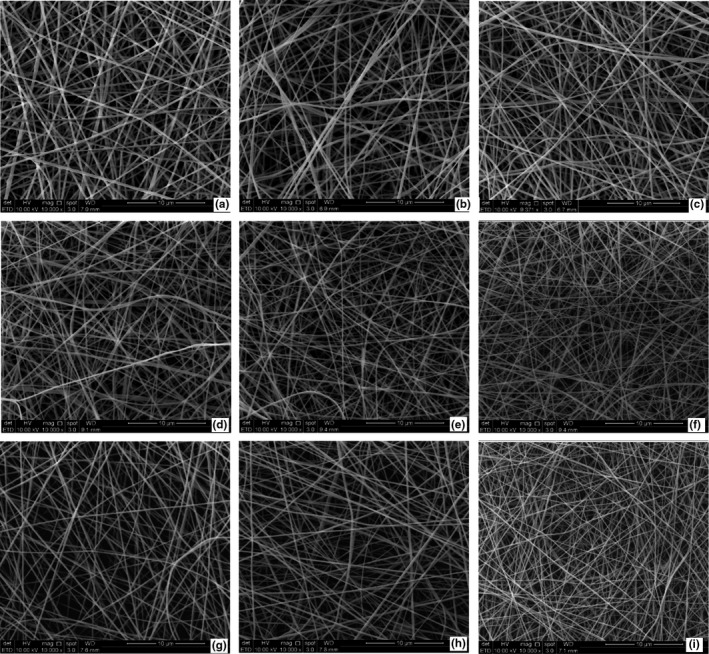
FESEM micrographs of electrospun gelatin nanofibers at 10,000×: cinnamaldehyde 1, 3, 5% (a–c), limonene 1, 3, 5% (d–f), eugenol 1, 3, 5% (g–i)

FESEM micrographs of all the electrospun fibers are presented in Figure 1. The spinning dope properties directly determine the spinning behavior and diameter of the resultant fibers in the mats (Haider et al., 2018). The fibers were smooth, cylindrical, and round in gelatin mats with all the BAs. The average fiber diameter in mats with CEO, LEO, and EEO were 358–441 nm, 351–446 nm, and 401–468 nm, respectively. Thicker nanofibers were obtained in the presence of 5% BAs. The lowering of total charges (i.e., conductivity) of the gelatin dope and lesser effect of Coulombic forces on gelatin chains might have reduced chain entanglement and hindered the extrusion of uniform nanofibers in the presence of higher concentrations of BAs and resulted in nonhomogenous fibers (Anu Bhushani & Anandharamakrishnan, 2014). BAs in zein resulted in higher heterogeneity as regards average fiber diameter (Altan et al., 2018).

### 3.3 Chemical structure analysis by ATR‐FTIR

**FIGURE 2:**
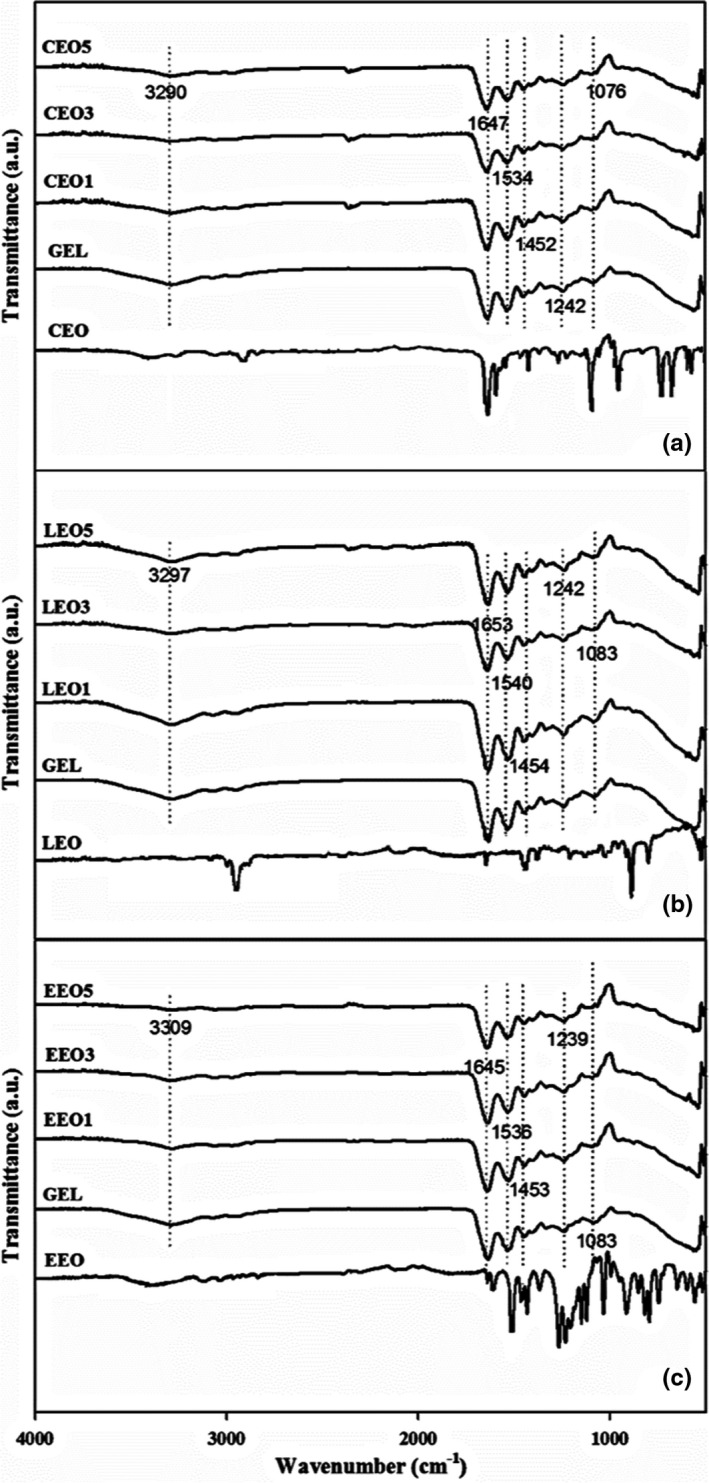
ATR‐FTIR spectra of electrospun gelatin mats with (a) cinnamaldehyde (CEO), (b) limonene (LEO), and (c) eugenol (EEO)

In electrospun mats, the chemical structure of gelatin and interaction between gelatin polypeptide chains and BAs were estimated by attenuated total reflectance Fourier transform infrared spectroscopy (ATR‐FTIR). The mats with different BAs presented somewhat similar spectra for major bands (Figure 2). The peak at 3297 cm^−1^ refers to the amide‐A group in gelatin indicating ‐NH stretching vibrations. A minor peak at 3078 cm^−1^ refers to the amide‐B group, represented by the stretching vibrations of ‐CH and N‐H. At 1647 cm^−1^, the presence of carbonyl stretching (C = O) along with in‐phase bending vibrations of the N‐H bond refers to the amide‐I group in gelatin.

The amide‐II band at 1540 and 1452 cm^−1^ is credited to the N‐H bend and C‐N stretching deformations. The small peak at 1242 cm^−1^ is a complex peak and is attributed to the N‐H bending and C‐N stretching, labeled as amide‐III (Chen et al., 2019). The peaks around 1612 and 1652 cm^−1^ correspond to the skeletal vibrations of C = C in benzene unsaturation and carbonyl moiety (C = O) in BAs, respectively. Moreover, peaks from 1000 to 1500 cm^−1^ and 750 to 900 cm^−1^ in the spectrum of CEO correspond to the C‐H bending of the benzene ring (Wu et al., 2017). Eugenol (EEO) spectra presented signature peaks in the range of 720–1250 cm^−1^ for the CH bending of aromatic groups. However, for the stretching of aromatic benzene moiety (C = C), sharp peaks were seen at 1636, 1606, and 1510 cm^−1^ (Somsap et al., 2019). Limonene (LEO) peaks appeared at 886, 1379, 1442, and 1646 cm^−1^ showing C = C, CH_3_ symmetric bending, CH_2_ bending, and C = C stretching of the exocyclic bondings, respectively. Other peaks observed at 2845 and 2961 cm^−1^ depict the antisymmetric and symmetric stretching of Sp^2^ and Sp^3^ in CH groups (Lin et al., 2018). Overall, the main peaks of the added BAs were masked by the major and high amplitude shoulders of the fish gelatin. This masking of bioactive compounds in the gelatin matrix signifies the successful encapsulation within nanofibers. The masking of BA signature peaks was also reported previously when chitosan and gelatin mats were added with various BAs (Haghighi et al., 2019). However, no new major peak appeared in the spectra, indicating no new covalent interaction, such as crosslinking, developed between the gelatin molecules and BAs. Hydrogen bonding and hydrophobic interaction between gelatin chains and various BAs have been indicated in the literature (Li et al., 2021).

### 3.4 Differential scanning colorimetry (DSC)

**FIGURE 3:**
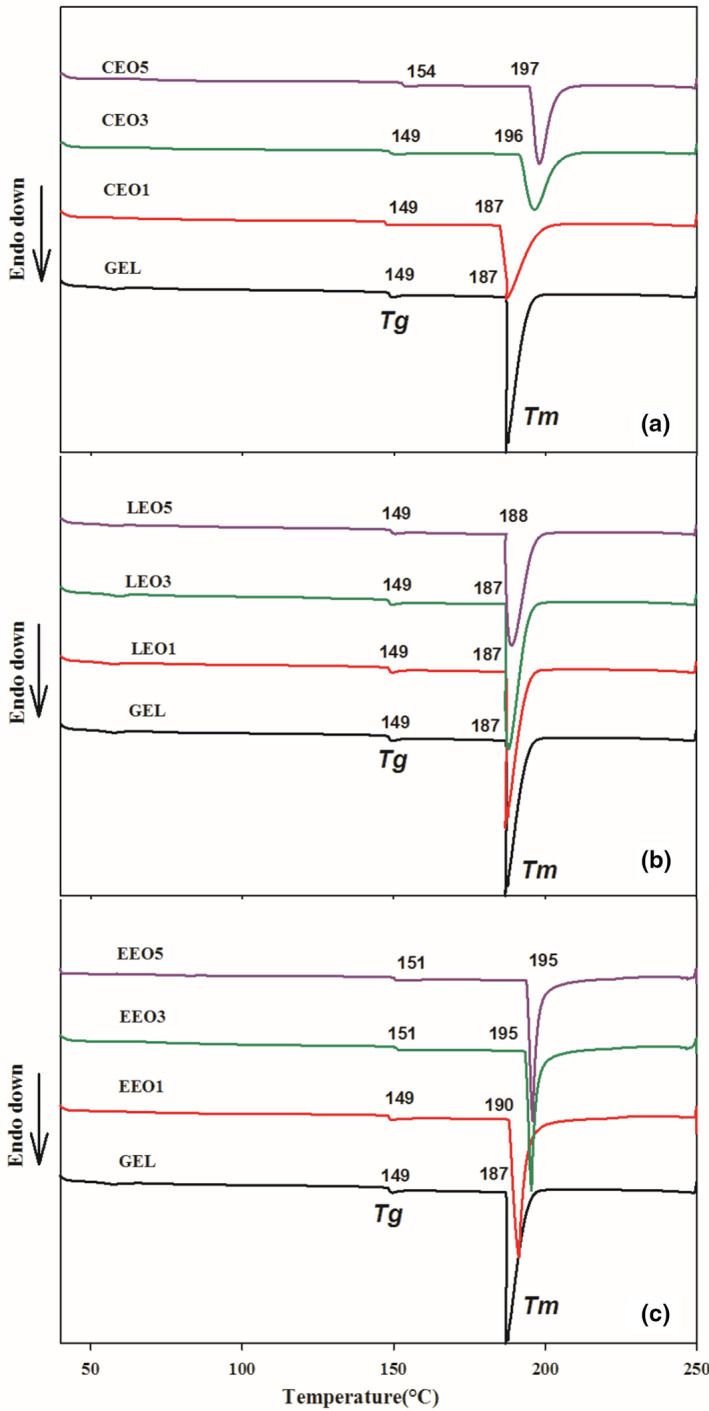
Glass transition (*T*_g_) and melting temperature (*T*_m_) of electrospun gelatin mats with (a) cinnamaldehyde (CEO), (b) limonene (LEO), and (c) eugenol (EEO)

DSC thermograms of electrospun gelatin mats are presented in Figure 3. All the thermograms of electrospun gelatin presented two clear endothermic peaks, indicating the glass transition temperature (*T*_g_) and melting temperature (*T*_m_). *T*_g_ is a second‐order transition, which represents the chain mobility of the amorphous polymeric regions under heat; however, *T*_m_ is correlated with the melting or destruction of partial crystallites in polymeric structures held by various interactions and is considered as a first‐order transition. Thermal analysis could be a good estimation of the successful encapsulation of BAs within the polymeric matrix (Feyzioglu & Tornuk, 2016).

In the case of *T*_g_, all the mats presented a temperature range between 149 and 154°C. The presence of BAs raised the *T*_g_ of gelatin mats; especially with a higher level of BAs, the temperature increased from 2 to 5°C. The improved chain‐to‐chain interactions in the presence of BAs could be a plausible explanation of the improved *T*_g_. Li et al. (2021) also observed an increase in the *T*_g_ of gelatin nanofibers in the presence of eugenol due to the presence of hydrogen bonding. Contrarily, the gelatin mats with LEO could not present any change in *T*_g_. Generally, in protein‐based polymeric structures, the increased chain stiffness renders a rise in *T*_g_ because of intramolecular and intermolecular bondings (Tongnuanchan et al., 2016). Different BAs might have affected the alignment of gelatin molecular chains and changed the level of interaction and presented different *T*_g_.

Similarly, improved *T*_m_ was seen in the thermograms in the presence of BAs. The *T*_m_ of mats was in the range of 187–197°C where the highest value was noted for mats with 5% CEO, indicating a difference of 10°C than the pristine gelatin mat. This could be due to the favorable protein–protein and protein–BA interactions in the presence of BAs (Tongdeesoontorn et al., 2021). The degree and strength of interactions between gelatin functional groups and benzene ring or alkyl chains of the BAs showed significant change in the thermal properties of mats. Overall, the BAs improved the thermal properties of gelatin mats in the order of CEO>EEO>LEO. Tongnuanchan et al. (2016) reported improved thermal stability of gelatin mats after the addition of basil EO. Similarly, encapsulation of summer savory EOs in chitosan nanoparticles augmented the thermal stability of nanoparticles (Feyzioglu & Tornuk, 2016).

### 3.5 Mechanical properties

TS is an indicator of strength, while EAB corroborates the flexibility and deformability of the electrospun gelatin mats. In the stress (force) versus strain (deformation) curve, the slope represents the Young's modulus (Y) and the height of the curve indicates TS, while the point of maximum strain is an indicator of EAB. The mechanical properties of gelatin mats are tabulated in Table 2. The incorporation of BAs modified the mechanical properties of the mats by manipulating protein–protein interactions. The highest TS was seen for the mats with LEO (1.581 MPa) followed by the CEO (1.148 MPa) and EEO (1.327 MPa). The nonpolar nature of the LEO, compared with other BAs, might have hindered the moisture plasticization of gelatin mats during the conditioning and resulted in greater TS.

**TABLE 2:** Mechanical properties of electrospun gelatin mats with different bioactive agents

Sample	Conc. (%)	Thickness (mm)	Tensile strength (MPa)	Elongation at break (%)	Young's modulus (Y) (MPa)
Control	‐	0.51 ± 0.03^a^	1.472 ± 0.112^ab^	45.14 ± 3.2^a^	11.029 ± 0.79^b^
CEO	1	0.49 ± 0.01^a^	1.148 ± 0.074^c^	36.27 ± 1.2^b^	7.194 ± 0.16^e^
	3	0.48 ± 0.01^a^	1.135 ± 0.084^c^	37.42 ± 1.3^b^	7.212 ± 0.45^e^
	5	0.50 ± 0.02^a^	1.124 ± 0.112^c^	38.04 ± 2.1^b^	7.304 ± 0.30^e^
LEO	1	0.48 ± 0.02^a^	1.581 ± 0.105^a^	27.15 ± 1.3^c^	11.998 ± 0.45^a^
	3	0.48 ± 0.01^a^	1.561 ± 0.088^a^	28.06 ± 1.7^c^	12.321 ± 0.20^a^
	5	0.49 ± 0.03^a^	1.574 ± 0.040^a^	28.74 ± 2.1^c^	12.402 ± 0.36^a^
EEO	1	0.48 ± 0.02^a^	1.327 ± 0.084^b^	43.90 ± 1.5^a^	10.012 ± 0.30^c^
	3	0.49 ± 0.01^a^	1.326 ± 0.074^b^	44.81 ± 2.4^a^	9.411 ± 0.45^cd^
	5	0.49 ± 0.01^a^	1.318 ± 0.123^b^	44.08 ± 2.0^a^	9.252 ± 0.20^d^

Note

Mean values with same letters are nonsignificant (*p* <.05).

Abbreviations: CEO, cinnamaldehyde; EEO, eugenol; LEO, limonene.

The mats with EEO presented the highest EAB (44.08%), suggesting greater flexibility. The least elasticity (EAB 27.15%) of mats with LEO is related to the highest TS. However, surprisingly, the lower concentrations of BAs (i.e., 1%) provided significant change in the tensile property while an increase in the concentration of BAs (i.e., 5%) could not bring a dose‐dependent increase in the observed properties. This type of mechanical behavior could be related to the saturation effect at gelatin/BA interface, where an increase in the concentration of BAs above a certain level did not result in any further change in tensile properties of gelatin mats. In line with current results, Said and Sarbon (2020) reported an increase in elongation but a reduction in the tensile strength and modulus of gelatin films with the addition of turmeric extract.

The mechanical properties of biodegradable polymeric mats are greatly dependent on the type and concentration of BAs added and the specific interactions existing at the polymer/BA interface (Tongnuanchan et al., 2016; Said & Sarbon, 2020). Wu et al. (2017) observed that the addition of CEO lowered the mechanical strength and elongation of gelatin mats. The authors suggested that the addition of CEO rearranged the protein matrix in such a way that it impaired polypeptide interactions.

### 3.6 3.6Antioxidant activity of mats

#### 3.6.1 Free radical scavenging activity

RSA (%) of gelatin mats was in the range of 63.86%–89.33% (Table 3). Among the three BAs, the highest RSA (89.33%) was observed for EEO at 5%. The observed RSA% for mats with added BAs was in the order of EEO>CEO>LEO. Nonetheless, the rise in the level of BAs from 1% to 5% could not depict a dose‐dependent shift in the RSA of the electrospun mats. Only the addition of carvacrol at 20%, but not at 5% and 10%, resulted in a significant change in the RSA of electrospun zein films (Altan et al., 2018).

**TABLE 3:** Antioxidant activity and encapsulation efficiency of mats with bioactive agents

Sample	Conc. (%)	Radical scavenging activity (%)	FRAP (μmol eq Fe^+2^)	Encapsulation efficiency (%)
Control	0	‐	‐	‐
CEO	1	64.89 ± 3.10^c^	1147.0 ± 15.0^g^	74.22 ± 0.76^f^
	3	67.69 ± 5.20^bc^	1275.3 ± 18.5^e^	82.32 ± 0.94^d^
	5	73.50 ± 6.01^b^	1337.33 ± 12.6^d^	86.63 ± 0.64^b^
LEO	1	46.04 ± 2.50^d^	912.0 ± 18.1^i^	71.83 ± 0.85^g^
	3	49.00 ± 3.20^d^	1015.6 ± 20.8^h^	80.04 ± 0.97^e^
	5	51.20 ± 5.09^d^	1186.66 ± 16.8^f^	93.27 ± 0.78^a^
EEO	1	84.34 ± 2.81^a^	1481.3 ± 14.5^c^	79.41 ± 0.65^e^
	3	87.93 ± 2.05^a^	1570.0 ± 15.7^b^	84.26 ± 0.71^c^
	5	89.37 ± 1.8^a^	1634.6 ± 22.81^a^	87.94 ± 1.03^b^

Note

Mean values with same letters are nonsignificant (*p* < .05).

Abbreviations: CEO, cinnamaldehyde; EEO, eugenol; LEO, limonene.

The observed pattern of RSA% is very true to the chemical structure‐based antioxidant activity of the BAs. The phenolic group in the molecule of EEO resulted in better RSA compared with CEO (aldehydes) and LEO (terpenes). In previous report, gelatin mats added with lavender and oregano EOs presented lower RSA%, which was correlated with the interactions between EOs and gelatin chains (Martucci et al., 2015).

#### 3.6.2 Ferric reducing antioxidant power (FRAP) assay

The addition of BAs in the gelatin mats resulted in significant improvement in the FRAP values (Table 3). In line with the RSA data, gelatin mats with higher concentrations of BAs (i.e., 5%) showed better antioxidant activity. Similarly, the overall antioxidant activity pattern of mats was in the order of EEO>CEO>LEO. The highest FRAP value was 1685.3 μmol eq Fe^+2^ in mats with 5% EEO, while the least activity (929.1 μmol eq Fe^+2^) was observed in mats with 1% LEO. In other words, EEO has the strongest reducing capacity and 1 g of the mat can change 1685 μmol of Fe^+3^‐TPTZ to Fe^+2^‐TPTZ. The better FRAP activity of EEO than others could be correlated with the presence of the phenolic group, which is a well‐known reducing agent. In a study, the FRAP values of tuna‐skin gelatin and bovine‐hide gelatin mats incorporated with rosemary and oregano extracts were measured. The oregano extract showed better antioxidant activity due to the phenolic group in its structure compared to films with rosemary (Gómez‐Estaca et al., 2009).

### 3.7 Encapsulation efficiency

The developed gelatin mats presented good encapsulation of BAs. However,a significant rise in EE% of mats was noticed with increasing the concentrationof BAs from 1% to 5% (Table 3). The EE% of gelatin mats for CEO, LEO, and EEO were in the range of 73%–85%, 71%–92%, and 78%–87%, respectively. LEO presented the highest encapsulation indicating better retention of the added level of LEO during electrospinning. Nonpolar amino acids in the gelatin polypeptide structure interact hydrophobically with the less polar or nonpolar functional groups of BAs (Nesterenko et al., 2013). Overall, high EE% of electrospun gelatin could be due to the faster solidification of the spinning dope jet into fiber during the short time (fraction of a second) of flight from the spinneret tip to the collector. Torkamani et al. (2018) also reported a higher EE% of electrosprayed gelatin nanoparticles in a phenolic‐rich bitter gourd extract.

### 3.8 In vitro antimicrobial activity

#### 3.8.1 In vitro antibacterial activity

BAs are well‐known natural antimicrobial agents due to their chemical structure and diverse functional groups. The disk diffusion method was adopted to assess the antibacterial activity of gelatin mats against a gram‐positive bacterium (*S*. *aureus*) and a gram‐negative bacterium (*E. coli*). The growth inhibition zone (mm) was significantly enhanced in the presence of BAs, and a greater inhibition was observed for mats with 5% BAs. However, CEO at 5% presented the highest growth inhibition of both *S*. *aureus* and *E. coli*. The least inhibition activity was seen for the mats with LEO, whereas the activity in EEO was comparable to that of CEO (Table 4). The least activity of mats with LEO could be correlated with its lower solubility in the agar media and least polarity (Yao et al., 2017).

**TABLE 4:** Antibacterial and antifungal activity (inhibition zone) of mats with bioactive agents

Sample	Conc. (%)	*E. coli* inhibition (mm)	*S. aureus* inhibition (mm)	*A. niger* inhibition (mm)
Control	0	‐	‐	‐
CEO	1	10.00 ± 0.50^ef^	12.83 ± 0.76^b^	9.50 ± 0.50^d^
	3	12.63 ± 0.55^bc^	13.50 ± 1.32^b^	12.20 ± 0.20^ab^
	5	14.33 ± 1.04^a^	18.83 ± 0.28^a^	13.10 ± 0.30^a^
LEO	1	8.33 ± 0.57^g^	9.33 ± 0.57^d^	8.40 ± 0.30^e^
	3	11.00 ± 1.0^de^	11.00 ± 1.64^cd^	11.00 ± 0.50^c^
	5	13.00 ± 1.0^abc^	12.00 ± 1.00^bc^	12.50 ± 0.30^ab^
EEO	1	8.66 ± 0.57^fg^	10.33 ± 0.57^cd^	8.60 ± 0.60^de^
	3	12.00 ± 1.0^cd^	12.83 ± 1.04^b^	11.80 ± 0.20^b^
	5	13.66 ± 1.15^ab^	13.33 ± 0.57^b^	12.80 ± 0.20^a^

Note

Mean values with same letters are nonsignificant (*p* < .05).

Abbreviations: CEO, cinnamaldehyde; EEO, eugenol; LEO, limonene.

Although mats with a higher concentration of BAs better inhibited both *S*. *aureus* and *E. coli*, a higher inhibition zone was observed for the former. The greater surface area of electrospun gelatin mats might have manipulated the release of BAs and maintained the critical inhibitory concentration at equilibrium, which impeded the bacterial growth, typically at higher loading of BAs. However, the lack of comparable antibacterial activity of the three BAs could be related to the different chemical structure, interaction with the gelatin, aqueous solubility, and release from the mats. Souza et al. (2014) reported the antiquorum sensing activity of BAs as an imperative mechanism in reducing the virulence and pathogenicity of the bacteria.

The lower inhibition of *E. coli* by gelatin mats with BAs could be correlated with the cellular structure of the bacteria. *E. coli* possesses a hydrophilic outer membrane with periplasmic spaces containing detoxifying enzymes, which ultimately neutralize the antimicrobial agents. Moreover, the presence of an outer thick lipopolysaccharide membrane in *E. coli* might have controlled the penetration of BAs and reduced the damage to the cell membrane and mitochondrial membrane (López et al., 2005). Similarly, Yanwong and Threepopnatkul (2015) reported lower antibacterial activity of gelatin mats added with peppermint and citronella extracts against *E. coli* compared with *S*. *aureus*.

#### 3.8.2 In vitro antifungal activity

The growth inhibition data of *A*. *niger* against the mats with three BAs are presented in Table 4. *A*. *niger* generally causes a disease called black mold and is a common contaminant of many fruits, vegetables, and bakery items. It also produces mycotoxins named as ochratoxins. Among the three BAs, mats with CEO presented the highest antifungal activity followed by mats with EEO. The least active mats were with the LEO (1%); however, a comparable antifungal activity was noticed at a higher concentration (5%). Overall, with an increase in the concentration of BAs in electrospun mats, a stronger fungal inhibition was observed with bigger inhibition zones.

Antifungal activity of the gelatin mats could be due to the disruption of fungal cell membrane and the associated enzymes by the BAs. The carbonyl functional group in CEO binds cell membrane proteins and damages the cell membrane (Altan et al., 2018; Burt, 2004). In contrast, EEO interrupts the biosynthesis of ergosterol in the cell membrane and destroys its integrity (Burt, 2004). Lucini et al. (2006) proposed fungal mycelial growth inhibition by phenols and terpenes by the excessive production of free radicals such as alkoxyl, hydroxyl, and alkoperoxyl radicals resulting in cell death. The observed variations in the antifungal activity of gelatin mats with BAs could be related to their chemical structures, the ratio of BAs to carrier polymeric matrix, and mutual interaction between the BAs and gelatin that ultimately influenced the diffusion of the active compound in the culture media to combat fungal growth.

### 3.9 Functional stability of gelatin mats

**FIGURE 4:**
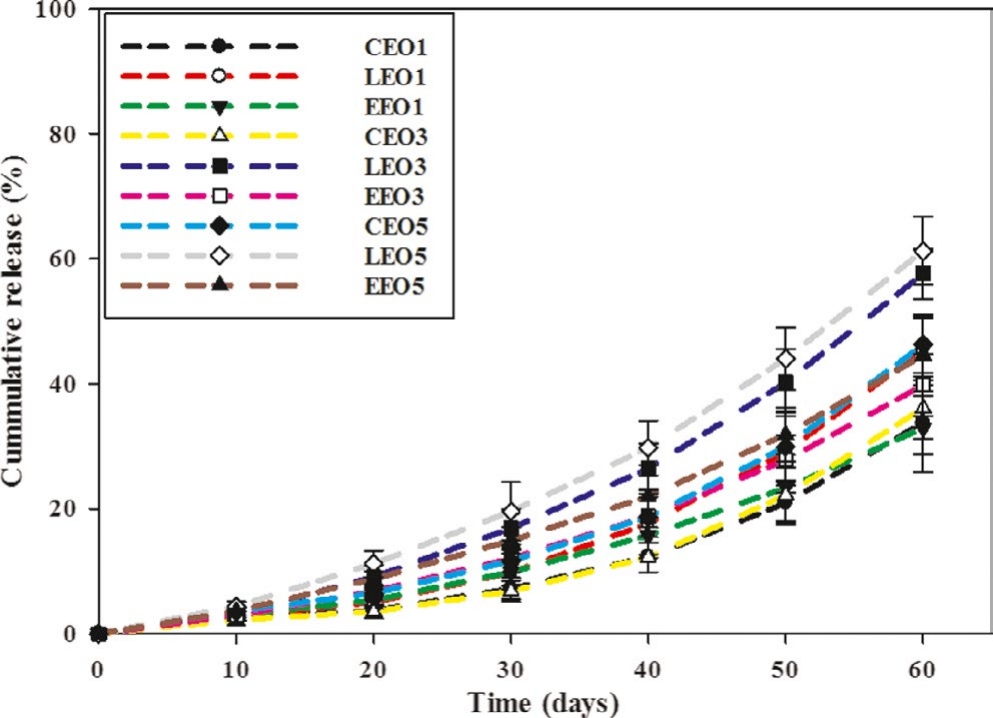
Cumulative release (%) of bioactive agents (BAs) from electrospun gelatin mats

The functional stability data of electrospun gelatin mats are presented as cumulative release of the BAs (Figure 4). The release of BAs was higher in LEO than in CEO and EEO. Mats with 1% BAs showed a cumulative release of 33.8%, 45.50%, and 32.8% for CEO, LEO, and EEO, respectively, at 60 days. However, when the concentration of BAs was raised to 5%, the cumulative release increased, i.e., 46.2%, 59.3%, and 44.5% for CEO, LEO, and EEO, respectively. The hydrophilic nature of gelatin might have favored the retention of more polar BAs (i.e., CEO and EEO) than LEO. Somsap et al. (2019) also reported a greater release of antimicrobial agents with increasing concentration to 10% from electrospun films of cellulose acetate/chitosan/gelatin. The longer storage time (60 days) might have resulted in higher moisture plasticization of hydrophilic gelatin mats, which increased chain mobility and facilitated the higher release of BAs. All electrospun mats presented somewhat similar cumulative release profiles under the experimental conditions irrespective of the type of BAs. However, lower release percentage of BAs at the start (10 days) suggests their slow release from the electrospun gelatin mats.

### 3.10 Bread preservation study

Freshly baked bread is perishable and only has a shelf life of 3–4 days (Jideani & Vogt, 2016). The major cause of bread spoilage is microbial growth, especially molds whose proliferation can be controlled by refrigeration. Nonetheless, the low temperature deteriorates bread acceptability caused by starch retrogradation called “staling.” In the bakery industry in UK, up to 3% losses are caused by molds, with annual spoilage of 70,000 tons of bread worth almost ₤60 million (Legan, 1993; Van Long et al., 2016). Thus, to minimize the bread spoilage, electrospun gelatin mats as antimicrobial active packaging are tested in this study. The inhibition of total aerobic bacteria and yeast and molds in bread was estimated at 10 days of storage. With increasing the concentration of BAs from 1% to 5%, a significant (*p* < .05) inhibition of aerobic bacteria was observed (Table 5). The highest inhibition was noticed for mats with 5% CEO, with the inhibition of 81%. Conversely, the least efficient inhibition (45%) was depicted by mats with 1% LEO. This bacterial inhibition trend supported the in vitro antibacterial data.

**TABLE 5:** Inhibition of total aerobic bacteria and yeast and molds by gelatin mats in wheat bread

Sample	Conc. (%)	Aerobic bacteria inhibition (%)	Yeast and mold inhibition (%)
CEO	1	53.0 ± 3.5^d^	43.0 ± 4.3^e^
	3	69.0 ± 2.4^b^	48.1 ± 5.0^d^
	5	81.0 ± 5.0^a^	56.5 ± 3.9^b^
LEO	1	45.0 ± 4.5^f^	40.8 ± 2.9^ef^
	3	55.0 ± 1.9^d^	46.3 ± 3.5^de^
	5	63.0 ± 3.0^c^	53.2 ± 4.0^bc^
EEO	1	50.0 ± 3.0^e^	42.5 ± 1.9^ef^
	3	61.0 ± 2.9^c^	52.5 ± 3.5^c^
	5	69.0 ± 5.0^b^	61.5 ± 5.0^a^

Note

Mean values with same letters are nonsignificant (*p* < .05).

Abbreviations: CEO, cinnamaldehyde; EEO, eugenol; LEO, limonene.

In the case of yeast and molds, mats with 5% EEO provided the best control over growth with 61% inhibition. In line with the antibacterial data, mats with LEO remained the least potent in hindering fungal proliferation. No fungal growth was noticed on the surface of the crumbs when stored with electrospun gelatin mats embedding 5% BAs, corroborating the effectiveness of mats as antifungal active packaging. The gelatin mats with different BAs presented variable inhibition of the microbial growth. These variations in the antimicrobial activity of mats could be due to different chemical structure, polarity and hydrophobicity of the added BAs. Altan et al. (2018) employed carvacrol (20%)‐loaded zein nanofibers for packaging of whole wheat bread and reported 99.6% and 87.6% inhibition of yeast and molds and aerobic bacteria, respectively, after 7 days of storage. The results suggest that the electrospun mats with BAs could extend the shelf life of preservative‐free wheat bread.

## 4 CONCLUSION

In this study, three BAs were embedded within the gelatin mats by electrospinning. The incorporation of BAs in gelatin spinning dopes modified conductivity, surface tension, and viscosity differently, which resulted in variations in the diameter of the nanofibers. The thermal stability of the mats was improved in the presence of BAs. However, the mechanical strength and flexibility of mats were impaired in the presence of BAs, except for LEO. Gelatin mats with EEO provided the best antioxidant properties, while mats with CEO excelled in antimicrobial activities against bacterial and fungal strains. The slower release of BAs validated the stability and suitability of the electrospun gelatin matrix in the retention of BAs over a sufficiently long period. Moreover, the absence of fungi on preservative‐free bread crumbs holds promise for the application of electrospun mats as antimicrobial active packaging for low‐to‐intermediate moisture foods.

## CONFLICT OF INTEREST

The authors have no relevant financial or nonfinancial interests to disclose.

## ETHICAL APPROVAL

This study does not involve any human or animal testing.

## Data Availability

The datasets generated during and/or analyzed during the current study are available from the corresponding author upon reasonable request.
